# Interchain disulfide engineering enables the efficient production of functional HLA-DQ-Fc fusion proteins

**DOI:** 10.1016/j.jbc.2024.107652

**Published:** 2024-08-08

**Authors:** Xiamuxiya Aisihaer, Hongjie Guo, Chang Liu

**Affiliations:** Antiger Therapeutics Inc., St Louis, Missouri, USA

**Keywords:** human leukocyte antigen, HLA-DQ, class II HLA, disulfide bond, Fc fusion protein, antibody-mediated rejection, immunotherapy

## Abstract

HLA-DQ molecules drive unwanted alloimmune responses after solid-organ transplants and several autoimmune diseases, including type 1 diabetes and celiac disease. Biologics with HLA molecules as part of the design are emerging therapeutic options for these allo- and autoimmune conditions. However, the soluble α and β chains of class II HLA molecules do not dimerize efficiently without their transmembrane domains, which hinders their production. In this study, we examined the feasibility of interchain disulfide engineering by introducing paired cysteines to juxtaposed positions in the α and β chains of HLA-DQ7, encoded by *HLA-DQA1∗05:01* and *HLA-DQB1∗03:01* respectively. We identified three variant peptide-HLA-DQ7-Fc fusion proteins (DQ7Fc) with increased expression and production yield, namely Y19C-D6C (YCDC), A83C-E5C (ACEC), and A84C-N33C (ACNC). The mutated residues were conserved across all HLA-DQ proteins and had limited solvent exposure. Further characterizations of the YCDC variant showed that the expression of the fusion protein is peptide-dependent; inclusion of a higher-affinity peptide correlated with increased protein expression. However, high-affinity peptide alone was insufficient for stabilizing the DQ7 complex without the engineered disulfide bond. Multiple DQ7Fc variants demonstrated expected binding characteristics with commercial anti-DQ antibodies in two immunoassays and by a cell-based assay. Lastly, DQ7Fc variants demonstrated dose-dependent killing of DQ7-specific B cell hybridomas in a flow cytometric, complement-dependent cytotoxicity assay. These data support inter-chain disulfide engineering as a novel approach to efficiently producing functional HLA-DQ molecules and potentially other class II HLA molecules as candidate therapeutic agents.

Human leukocyte antigens (HLA) are membrane proteins that present antigens to T cell receptors to initiate adaptive immune responses (1). The genetic diversity of HLA molecules enables the presentation of a wide array of microbial peptides to T cells for infection control or endogenous neoantigens for cancer surveillance. However, the extreme polymorphism of HLA in the human population also marks the distinction between self and non-self, causing most individuals to be HLA-mismatched among each other (2). In allogeneic solid-organ transplantations, more HLA mismatches in a donor-recipient pair correlate with shorter graft survival, even under potent contemporary immunosuppression regimens (3, 4, 5). Moreover, exposure to mismatched foreign HLA often leads to alloimmunization and the formation of donor-specific antibodies (DSA), which are the primary cause of antibody-mediated rejections (AMR) (6, 7). Due to the lack of effective management for AMR, this form of rejection has emerged as a leading cause of graft failure (8). While DSA against both class I (HLA-A, -B, -C) and class II (HLA-DR, -DQ, -DP) antigens can cause clinically significant rejections, the latter, especially DSA against HLA-DQ, has been shown to particularly prevalent and detrimental (9, 10, 11, 12, 13, 14).

There has been an unmet medical need for patients with AMR due to the lack of any FDA-approved treatment for AMR. The current standard of care remains using plasma exchange to remove circulating DSA, coupled with intravenous immunoglobulin infusion (15, 16). Additional therapeutic agents targeting T cells, B cells, plasma cells, complement factors, and cytokine signaling may be used to lower the production of DSA or inhibit its effector functions (16, 17). Many of these therapies have been repurposed from treatments for cancers or autoimmune diseases. Although they demonstrated some benefits in certain AMR cases, their efficacy is yet to be established in randomized clinical trials (18, 19, 20, 21, 22, 23, 24). Moreover, none of these approaches can selectively target pathogenic DSAs or DSA-producing cells. Unfortunately, broad immunosuppression increases the risk of infections and cancers, which are the primary causes of mortality in the transplant patient population (25).

Mismatched donor antigens may guide the selective targeting of anti-HLA DSA or DSA-producing cells when incorporated in a biologic along with other components that confer effector functions. Recently, we generated class I HLA-Fc fusion proteins and demonstrated their ability to selectively deplete cognate B cell hybridomas *in vitro* and *in vivo* (26). The HLA portion of the biologic was a single chain trimer consisting of a high-affinity peptide, the β2-microglobulin, and the extracellular domains of class I HLA α chain (27, 28), which was followed by an Fc fragment at the C-terminal end. The HLA portion provided the desired selectivity, while the Fc portion mediated the complement-dependent cytotoxic killing of target cells (26). However, adopting this strategy to target class II HLA-specific DSA and antibody-producing cells remains a challenge due to the complexity of producing soluble class II HLA complexes (29, 30, 31). Notably, the extracellular domains of class II MHC α and β chains do not dimerize without their transmembrane domains (32). The two chains are also similar in size and assembled differently than the class I complex, limiting the wide adoption of the single-chain trimer approach (33).

In this study, we aimed to create a stable class II HLA-Fc fusion protein *via* disulfide engineering. We started with a common class II antigen, HLA-DQ7, present in 35.2% of the donor population in the United States and frequently targeted by DSAs in transplant patients (14). We screened a series of paired point mutations in α and β chains that may promote inter-chain disulfide bond formation. We identified several highly expressed and stable variants, examined their binding characteristics, and tested the feasibility of targeting DQ7-specific B cell hybridomas using these novel agents.

## Results

### Disulfide engineering of soluble peptide-HLA-DQ7-Fc (DQ7Fc) fusion protein

**Figure 1:**
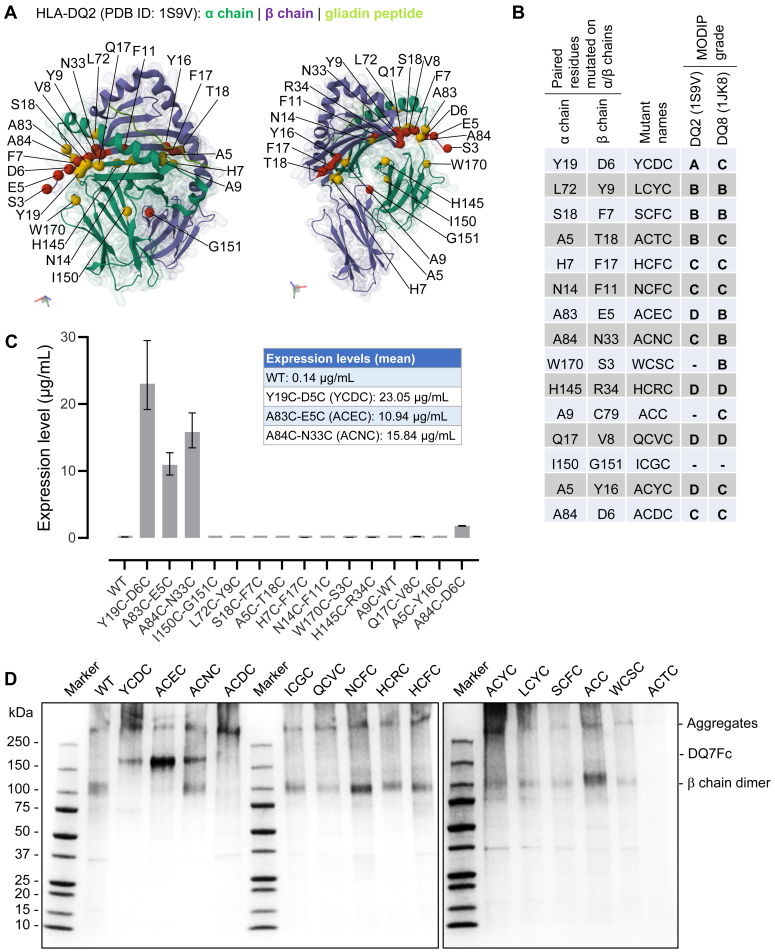
**Disulfide engineering of DQ7Fc fusion proteins.***A*, fifteen pairs of candidate positions for disulfide engineering were visualized on the structural model of HLA-DQ2 (PBD ID: 1S9V) from two angles (*left* and *right* panels). The α chain, β chain and gliadin peptide were colored in *purple*, *green*, and *lime*. Residues at candidate positions were represented by *red* spheres and *yellow* spheres on the α chain and β chain, respectively. *B*, Grades of fifteen pairs of candidate positions for disulfide engineering were predicted by the MODIP procedure based on HLA-DQ2 (1S9V) and -DQ8 (1JK8) models. Grade A is suitable for disulfide bond formation; grade B is geometrically acceptable but with some steric distortion; grade C is spatially too close for a disulfide bond; grade D is geometrically unfavorable. *C*, expression levels of wild type and disulfide-engineered DQ7Fc variants in the supernatant of CHO cells on day 7 after transfection, as quantified by ELISA. Means and 95% confidence intervals (CI) of the DQ7Fc concentrations interpolated from the standard curve were plotted. The mean concentrations were also reported in the inset table. *D*, wild type (WT) and disulfide-engineered DQ7Fc variants were purified from the supernatants by protein A chromatography and analyzed by SDS-PAGE under a non-reducing condition. Dimeric DQ7Fc, with an expected size of ∼150 kDa, was detected for the top three expressors, YCDC, ACEC, and ACNC.

To identify paired positions in HLA-DQ7 for disulfide engineering without its structure, we first analyzed existing models of HLA-DQ2 (PDB ID: 1S9V) (34) and -DQ8 (PDB ID: 1JK8) (35) by visual inspection and by the Modeling Of Disulfide bonds In Proteins (MODIP) procedure (36). Multiple paired positions from the α1/β1 domains of these heterodimers were adjacent to each other at the bottom or side of the peptide binding groove; we also found several candidate positions between the α2/β1 and α2/β2 domains (Fig. 1*A*). The MODIP procedure identifies candidate pairs of residues to be mutated to cysteines where covalent crosslinking by disulfide bonds can be accommodated without significant steric strain; the potential crosslinks are further graded as follows (36). Grade A indicates the modeled disulfide bond distance and dihedral angles are within accepted ranges; grade B indicates the bond is geometrically suitable but with some steric distortion; grade C indicates the sites are spatially too close for disulfide bond; grade D indicates that sulfurs cannot be fixed geometrically. The grades were unfavorable for most candidate positions, and results generated with the HLA-DQ2 and -DQ8 structural models were frequently in disagreement (Fig. 1*B*).

Next, we co-transfected DNA encoding paired α and β chains at a 2:1 ratio for the wild type and variant HLA-DQ7 into CHO cell lines and measured their expression in the supernatant. In addition to the paired mutations introduced for disulfide bond formation, the β chain sequence was preceded by sequences for the signal peptide, a placeholder CLIP peptide (PVSKMRMATPLLMQA), and a (GGGSG)_2_ flexible linker at the N-terminal end (Fig. S1). The C-terminal end of the β chain sequence was fused to the human IgG1 Fc fragment. A signal peptide preceded the α chain at the N-terminal end. With this design, we expected to generate a disulfide-stabilized peptide-HLA-DQ7 (DQ7) complex that further dimerizes *via* the Fc fragment to form a dimeric DQ7Fc fusion protein within an immunoglobulin-like framework. We observed minimal expression of the wild-type DQ7Fc in the supernatant as measured by ELISA. In contrast, the expression levels were significantly higher for three DQ7Fc variants, Y19C-D6C, A83C-E5C, and A84C-N33 C (abbreviated as YCDC, ACEC, and ACNC hereafter), with up to 165-fold increase over the wild type (Fig. 1*C*). The rest of the mutants exhibited minimal expression as the wild type.

We purified the wild-type and variant DQ7Fc proteins by protein A chromatography and evaluated them by sodium dodecyl sulfate-polyacrylamide gel electrophoresis (SDS-PAGE) (Fig. 1*D*). Under the non-reducing condition, variants YCDC, ACEC, and ACNC showed a distinct band of ∼150 kDa, consistent with the expected molecular weight of dimeric DQ7Fc. This band was absent for the wild type and other variants, while a smaller band of ∼100 kDa was observed with a size comparable to the β chain-Fc dimer without paired α chains. Under the reducing condition that disrupted interchain and intrachain disulfide bonds, we observed the β chain of ∼50 kDa across all variants; the α chain of ∼25 kDa was also observed with variable intensities (Fig. S2).

We optimized the expression conditions for YCDC and ACEC variants using a lower temperature (28 °C) and longer incubation time (11 days). The yield of YCDC increased from 10 mg to 23 mg per liter of cell culture at a purity of 60%, with apparent aggregates after protein A purification. In comparison, ACEC showed a lower yield (14 mg/L of culture) but improved purity (80%). Further purification of ACEC using Q Sepharose fast flow resin separated the major dimeric DQ7Fc from the minor aggregate and β chain-Fc dimer, increasing the purity of ACEC to 90% by size exclusion chromatography. The above data together suggested that stable DQ7Fc variants can be generated by site-directed disulfide engineering to achieve increased expression levels and production yields.

### Characteristics of amino acid residues mutated for successful disulfide engineering

**Figure 2:**
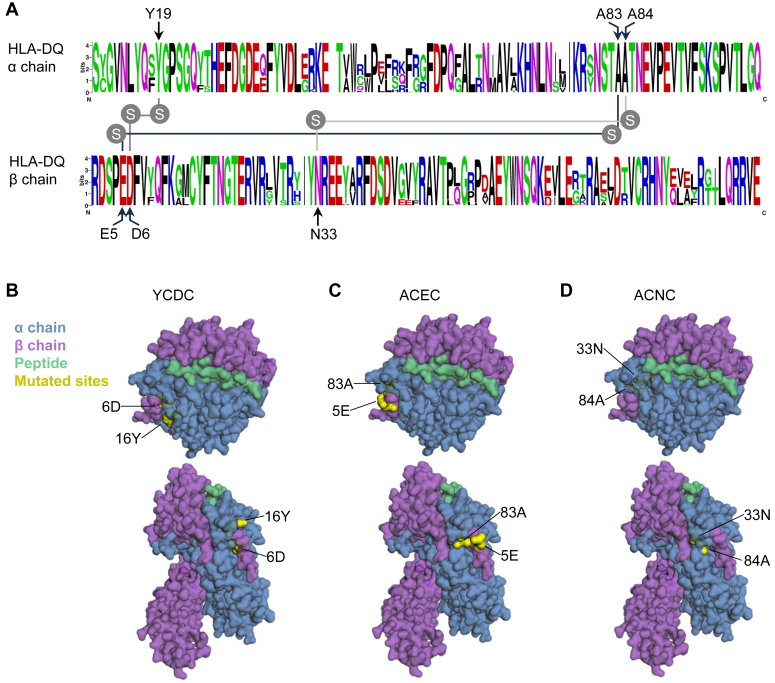
**Characteristics of sites mutated in stably expressed DQ7Fc variants.***A*, graphical alignment of protein sequences of all HLA-DQ α chains and β chains from the IPD-IMGT/HLA database were generated using weblogo (61); only the alignment for the α1 and β1 domains are shown. The three pairs of residues successfully mutated for disulfide engineering were annotated. These residues were conserved with no naturally occurring cysteine variants. *B–D*, the surface exposure of the mutated residues (*yellow*) in YCDC, ACEC, and ACNC variants were visualized on an HLA-DQ7 model from pHLA3D. The α chain, β chain and gliadin peptide were colored in *blue*, *purple*, and *green*. *Upper* panels are surfaces viewed from the *top* of the molecule, which interact with T cell receptors; *lower* panels are side views of these molecules.

The HLA protein sequences are extremely polymorphic in the human population, allowing the presentation of diverse peptides. The three pairs of amino acid residues successfully mutated for disulfide engineering were conserved among unique protein sequences of 337 HLA-DQA1 and 1516 HLA-DQB1 molecules in the IPD-IMGT/HLA database (version 3.54 released in October 2023) (37). Moreover, cysteine residues did not naturally occur at these positions in any DQ variants in humans (Fig. 2*A*).

Next, we visualized the solvent accessibility of these mutated residues, which may imply the potential of creating unwanted immunogenic epitopes by the mutations (Fig. 2, *B*–*D*). In an HLA-DQ7 model generated with the pHLA3D program (38), all three pairs of mutated residues in YCDC, ACEC, and ACNC were visible at the surface of the model. At side views, A83-E5 appeared to be more exposed than Y19-D6 and A84-N33. All three pairs were largely shielded by the α helix of the α chain when viewed from the top of the molecule and thus unlikely to be recognized by T cell receptors (TCR). For possible recognition by B cell receptors (BCR), we reviewed all 27 antibody-verified HLA-DQ eplets cataloged in the HLA epitope registry (39). None of these eplets on the α or β chain were in contact with the three pairs of residues mutated for disulfide engineering. While the modeling above indicates a limited immunogenic potential of the three paired mutations, further investigations in an alloimmunization model are warranted.

### Effect of the peptide on the DQ7Fc(YCDC) fusion protein

**Figure 3:**
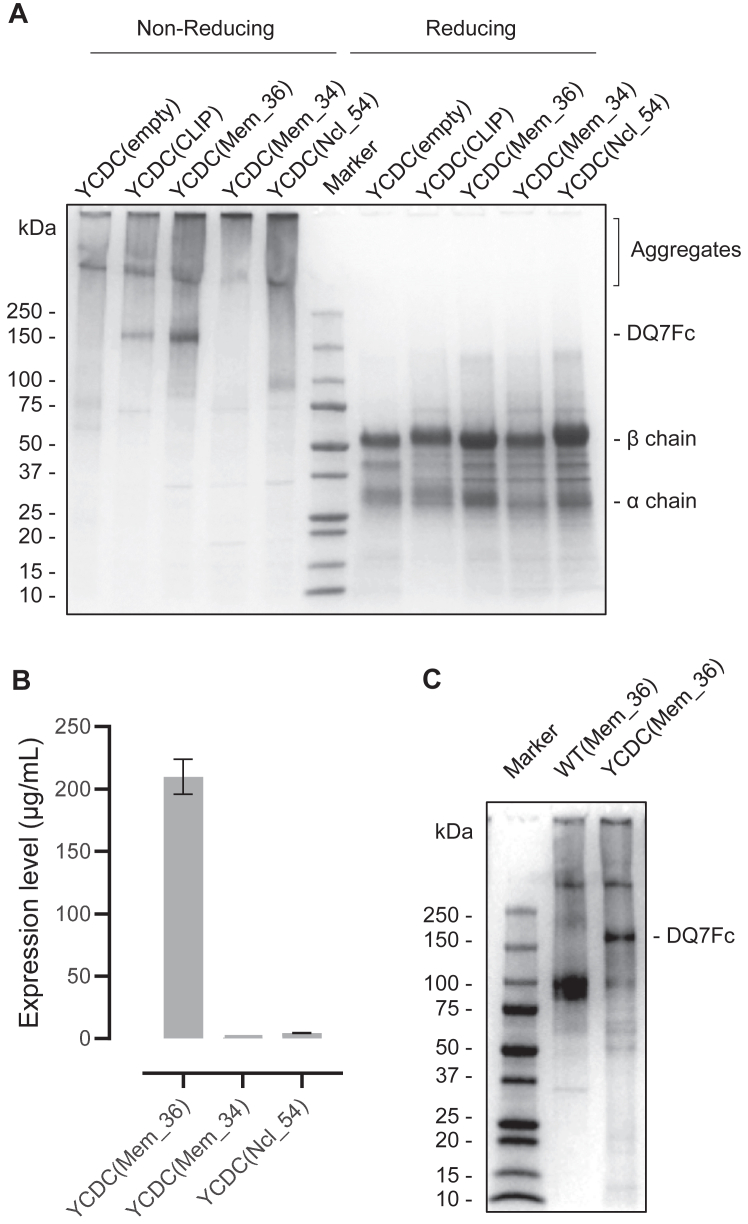
**Effects of peptides on the DQ7Fc(YCDC) variant.***A*, a disulfide-engineered dimeric DQ7Fc variant, YCDC, were produced without a linked peptide or with 4 different peptides, CLIP, Mem_36, Mem_34, and Ncl_54. The latter three were SARS-CoV-2-derived peptides known to bind to DQ7, with Mem_36 showing the highest affinity. All variants were purified from the supernatants by protein A chromatography and analyzed by SDS-PAGE under non-reducing and reducing conditions. Variants with an expected size of ∼150 kDa was only detected in the presence of CLIP or Mem_36. *B*, expression levels of three YCDC variants with SARS-CoV-2-derived peptides in the supernatant of CHO cells on day 7 after transfection, as quantified by ELISA. Means and 95% CI of the protein concentrations interpolated from the standard curve were plotted. C, wild type (WT) and YCDC variant linked with Mem_36 peptide were purified and analyzed by SDS-PAGE. Stable DQ7Fc was not detected without the interchain disulfide bond (WT).

We further examined whether the stability of one of the DQ7Fc variants, YCDC, is peptide-dependent. Without a linked CLIP to occupy the peptide binding groove, the DQ7Fc(YCDC) construct failed to express any detectable protein with the expected size (Fig. 3*A*), suggesting that endogenous peptides in the cultured cells were insufficient to stabilize the soluble protein. We searched the IEDB database (40) and found three SARS-CoV-2-derived peptides that bound to DQ7 at high affinities, defined as a half maximal inhibitory concentration (IC50) of <1000 nM in a radioligand competition binding assay (41). We next generated variants by replacing CLIP in the DQ7Fc(YCDC) protein with these peptides: Mem_36 (IC50 = 48 nM), Mem_34 (IC50 = 649 nM), and Ncl_54 (IC50 = 794 nM). Quantification of these variant proteins in the supernatant by ELISA showed that the expression level of the Mem_36 variant was 8-fold higher than that of the CLIP variant. At the same time, the expression of variants containing Mem_34 and Ncl_54 was minimal (Fig. 3*B*). The variant carrying the Mem_36 peptide also showed a stronger band than the variant carrying CLIP by SDS-PAGE; variants containing the other two SARS-CoV-2 peptides were undetectable (Fig. 3*A*). These data suggest that the stable expression of disulfide-engineered DQ7Fc is peptide-dependent, and the presence of a higher affinity peptide correlates with a higher expression and yield of the fusion protein. Despite the importance of high-affinity peptide in this context, DQ7Fc carrying Mem_36 without the interchain disulfide bond could not be detected (Fig. 3*C*), indicating a high-affinity peptide alone is insufficient for the stable expression of dimeric DQ7Fc.

### Binding characteristics of the DQ7Fc fusion protein

**Figure 4:**
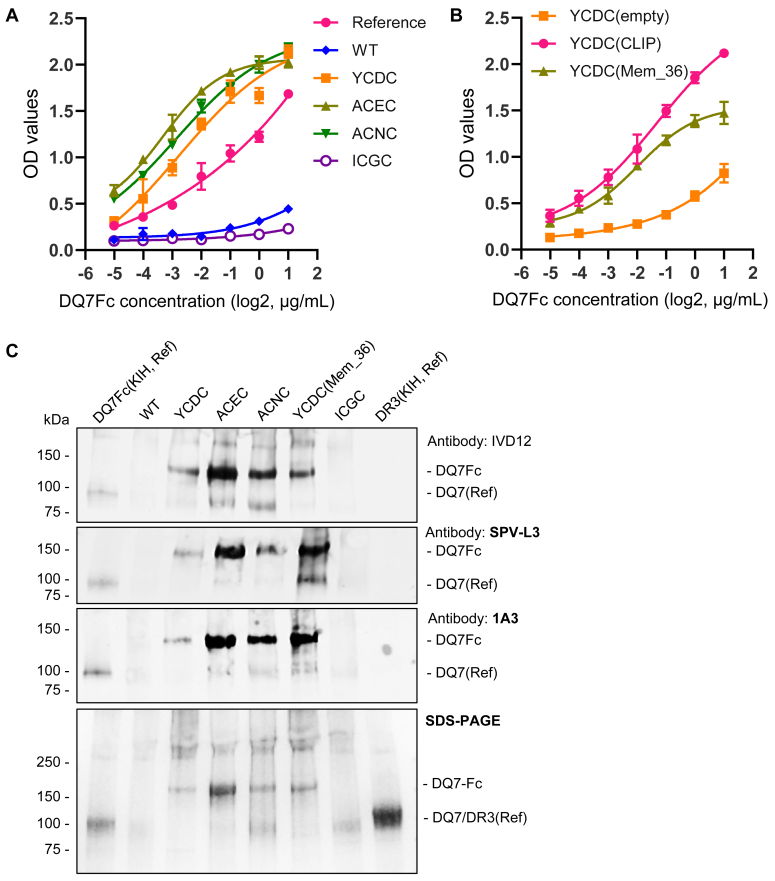
**Binding characteristics of DQ7Fc variants.***A*, reference monomeric DQ7Fc (reference; knob-into-hole or KIH), wild type (WT), and disulfide-engineered DQ7Fc variants (YCDC, ACEC, ACNC, ICGC) were tittered by serial 1:2 dilutions; their binding to anti-DQ7 antibody (IVD12) was assessed by ELISA. Means and standard deviations (SD) of the OD values were plotted with curve fitting by the four-parameter logistic (4PL) regression model. *B*, the binding of YCDC variants without a linked peptide or with a linked CLIP or Mem_36 to anti-DQ7 (IVD12) was assessed by ELISA as in *A*. *C*, reference monomeric DQ7Fc (KIH), wild type (WT), and disulfide-engineered DQ7Fc variants [YCDC, ACEC, ACNC, YCDC(Mem_36), ICGC] were analyzed by Western blot using three DQ antibodies, IVD12, SPV-L3, and 1A3 (*top* three panels). DR3(KIH) was included as a negative control that is not expected to be recognized by the DQ antibodies (*last* lane). The SDS-PAGE image was included as a loading control (*bottom panel*).

To examine whether DQ7Fc variant proteins were folded properly with intact surface epitopes, we measured their binding to a monoclonal anti-DQ7 antibody. As a control, we produced monomeric DQ7Fc as a reference protein using the knob-into-hole (KIH) technology, co-expressing the DQ7 α chain linked with Fc(knob) and the β chain linked with Fc(hole) in CHO cells (31). The heterodimerization of Fc(knob) and Fc(hole), typically used for producing bispecific antibodies, promoted the formation of the DQ7 complex in this case. Serial diluted YCDC, ACEC, and ACNC variants of DQ7Fc demonstrated similar binding profiles to anti-DQ7 (IVD12), as measured by ELISA (Fig. 4*A*). All three variants showed higher affinities than the reference protein. In contrast, the wild type and another low-expression DQ7Fc variant, ICGC, showed minimal binding. The YCDC variants, carrying CLIP and Mem_36 peptides, respectively, also shared similar binding profiles to anti-DQ7 (IVD12) and demonstrated higher affinities than the empty YCDC variant not carrying a peptide (Fig. 4*B*).

We further examined the binding of DQ7Fc variants with three anti-DQ antibodies (IVD12, SPV-L3, and 1A3) by Western blot. These antibodies have unique binding specificities: IVD12 recognizes the DQ7 subtype and the DQ3 parent type (specificity verified by the FlowPRA assay); SPV-L3 is specific to the DQ α chain; 1A3 is specific to the DQ β chain. All three antibodies detected the DQ7Fc(KIH) and 4 DQ7Fc variants tested, namely YCDC(CLIP), ACEC(CLIP), ACNC(CLIP), and YCDC(Mem_36), at the expected molecular weight (Fig. 4*C*). The wild type and ICGC variant proteins were not detected by any of these antibodies at the expected molecular weight. We also produced DR3-Fc(KIH) as an additional negative control, which was not detected by any of the DQ-specific antibodies (Fig. 4*C*). The above results suggest that multiple disulfide-engineered DQ7Fc variant proteins had intact surface epitopes recognized by antibodies of known specificities.

### Complement-dependent cytotoxicity effect of DQ7Fc on cognate B cell hybridomas

**Figure 5:**
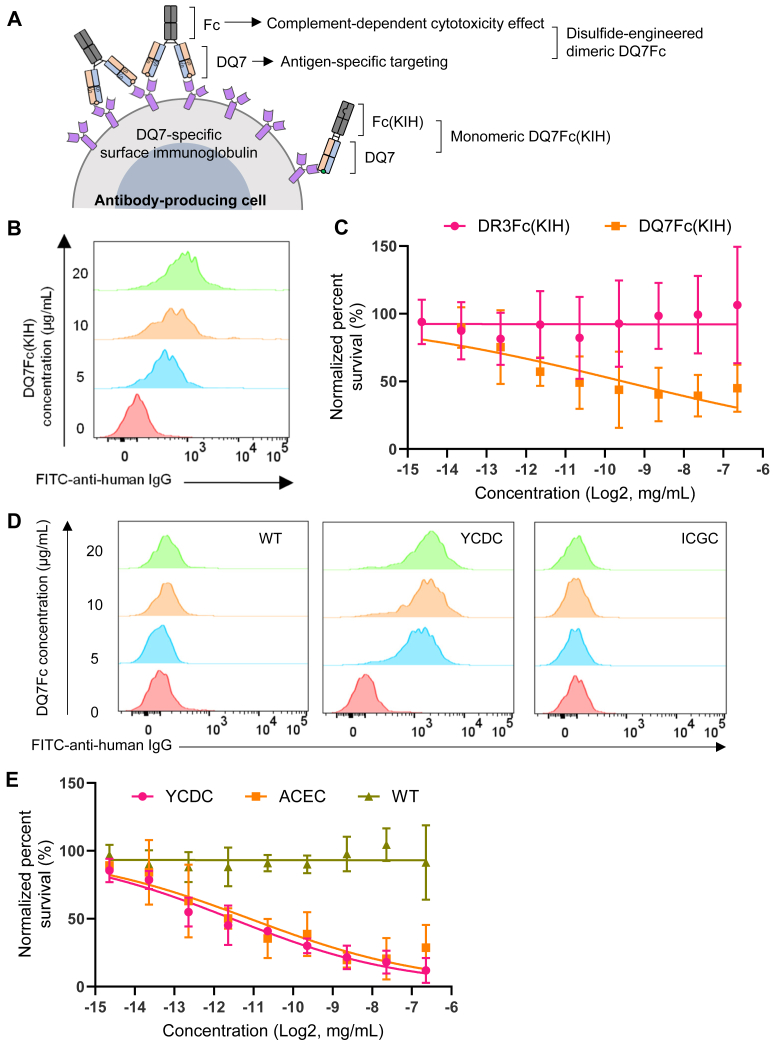
**Antigen-specific targeting of DQ7-specific B cell hybridomas by DQ7Fc variants.***A*, schematic of antigen-guided and complement-dependent cytotoxicity (CDC) on cognate antibody-producing cells mediated by disulfide-engineered dimeric DQ7Fc and monomeric DQ7Fc(knob-into-hole or KIH). *B*, binding of increasing amounts of monomeric DQ7Fc(KIH) to the DQ7-specific B cell hybridoma cells (IVD12) was measured by flow cytometry. Bound DQ7Fc, with a human IgG1, was detected by FITC-conjugated anti-human IgG. *C*, the killing of IVD12 cells by DQ7Fc(KIH) and DR3Fc(KIH) in the presence of rabbit complement was measured by a flow cytometric CDC assay. Means and SD of percent survival were plotted with curve fitting by nonlinear regression using a variable slope model. *D*, binding of increasing amounts of WT, YCDC, and ICGC DQ7Fc to IVD12 cells was measured by flow cytometry as in *B*. *E*, the killing of IVD12 cells by WT, YCDC, ACEC DQ7Fc in the presence of rabbit complement was measured by a flow cytometric CDC assay and results plotted as in *C*.

We hypothesized that KIH and disulfide-engineered DQ7Fc proteins could mediate antigen-specific killing of cognate antibody-producing cells such as the IVD12 B cell hybridomas. Mechanistically, the DQ7 antigens may guide the fusion protein to specific B cell receptors to enable Fc-dependent complement activation and cytotoxicity effect (Fig. 5*A*). As expected, DQ7Fc(KIH) bound to IVD12 cells and caused significant shifts in flow cytometric analysis (Fig. 5*B*). DQ7Fc(KIH) also killed IVD12 cells in the flow cytometric complement-dependent cytotoxicity (CDC) assay with an absolute CC50 of 22 μg/ml. In contrast, DR3Fc(KIH) did not kill IVD12 cells at high concentrations as a specificity control. IVD12 cells showed an increased baseline survival rate (36%) than the A2-specific PA2.1 cells and B7-specific BB7.1 cells (∼0%) we tested previously (26), which may be a property intrinsic to each hybridoma cell line. After adjusting for the background signal, the relative CC50 for DQ7Fc(KIH) was 1.3 μg/ml (Fig. 5*C*). Similar to the DQ7Fc(KIH) reference protein, we detected robust binding of DQ7Fc(YCDC) to IVD12 cells by flow cytometry while wild-type DQ7Fc and ICGC variant were not detected on the cells (Fig. 5*D*). We also detected significant killing of IVD12 cells by DQ7Fc(YCDC) and DQ7Fc(ACEC) in a dose-dependent manner (Fig. 5*E*), with absolute CC50 of 3 μg/ml and 4.7 μg/ml, respectively. After adjusting for the background in IVD12 cells, the relative CC50 values were 0.33 μg/ml and 0.46 μg/ml for YCDC and ACEC variants, respectively. These results demonstrate that disulfide-engineered dimeric DQ7Fc proteins can function as a targeting agent to mediate antigen-specific and complement-dependent killing of cognate cells.

## Discussion

This study demonstrated the feasibility of a novel approach to generating soluble dimeric DQ7Fc fusion proteins *via* disulfide engineering to stabilize the DQ7 complex. We identified multiple variants with increased expression levels and production yields compared to the wild type without disulfide engineering. These variants were functional and capable of antigen-guided killing of cognate antibody-producing cells. The approach may also apply to engineering other class II HLA molecules and hold the promise of improving the production of biologics containing class II HLA for immunotherapies of diverse indications.

Disulfide engineering offers ample opportunities as well as challenges in biomedical applications. The covalent disulfide bond occurs in natural proteins and is a logical approach to stabilizing recombinant proteins (42). Achieving the desired outcome would require sufficient information about the structural features of the molecule of interest, based on which a disulfide bond can be inserted between positions within a suitable distance without causing steric distortion. However, the rules governing disulfide bond formation relative to the overall protein stability have not been fully elucidated, and the engineered disulfide bond may have no stabilizing effect or even be destabilizing in some cases (43, 44). Although several bioinformatic tools have been developed to assist site selection for mutagenesis (45, 46, 47, 48), the success rates varied among tools and specific projects (48). Moreover, both false positive and false negative predictions have been reported. For false positives, mutations of predicted positions failed to form disulfide bonds, whereas, for false negatives, disulfide bonds were successfully engineered despite violating the geometric constraints of bioinformatic models (47, 49). We used MODIP to facilitate the disulfide engineering in this study with a success rate of 25% if counting all grade A or B predictions based on the DQ2 model (1S9V); the success rate was 40% based on the DQ8 model (1JK8). On the other hand, low-grade predictions (C or D) based on the two models would have missed two or one of the stable variants. The relatively low success rate of the predictions was expected, as a similar rate (33%) was reported previously for inter-molecular disulfide bond engineering based on a protein-protein interaction model (50).

The efficient production of disulfide-engineered DQ7Fc fusion proteins faced several additional hurdles. DQ7 may already contain multiple intrachain disulfide bonds within the α2, β1, and β2 domains like DQ2 and DQ8 (34, 35); the fused Fc portion included additional intrachain and interchain disulfide bonds. These existing disulfide bonds increased the complexity of introducing an additional disulfide bond between the DQ7 α and β chains. In light of the HLA diversity in humans, structural models were only available for a few HLA-DQ molecules but not DQ7. Nevertheless, disulfide-engineered dimeric DQ7Fc demonstrated expected molecular weight, recognition by known antibodies, and functional properties. These encouraging results indicated that the approach involving conserved positions among DQ molecules may be generalizable to class II HLA beyond DQ7. Finally, the peptide presented by the HLA dimer further complicated the engineering effort. Our data suggested that endogenous peptides in the cell culture could not sustain the expression and purification of stable DQ7Fc, while a placeholder CLIP or higher-affinity peptide linked to the β chain was required for this process. Notably, without the interchain disulfide bond, a higher-affinity peptide alone was insufficient for stabilizing the dimeric DQ7Fc protein. Given the effect of peptides on the yield of DQ7Fc, it will be beneficial to engineer a cleavable CLIP, which can be replaced by diverse peptides relevant to specific TCR in different diseases.

Our work adds to the existing tools for producing recombinant class II HLA proteins for various applications, and all carry unique pros and cons. First, leucine zippers, consisting of two alpha-helices that stably twist around each other through hydrophobic interactions between leucine residues, have been widely used to promote protein heterodimerization. Most class II HLA or MHC tetramers for labeling specific TCR are routinely made with leucine zipper tails (51, 52). Leucine zippers are structural motifs primarily found in transcription factors to regulate their DNA binding. As parts of intracellular proteins, their immunogenic potentials remain uncertain (53) and could be recognized as foreign in the extracellular space. Second, class II HLA or MHC complex can also be produced as single-chain trimers (33, 54, 55, 56, 57) with the advantage of expressing all components from one combined coding sequence. However, a relatively long flexible linker, such as T(SGGGG)_4_SSS (33, 56), is required to connect the C terminal end of the β chain to the N terminal end of the α chain. The distance ranges from 45 to 47 Å as measured in the DQ2 and DQ8 models (1S9V, 1JK8). The reported yield was approximately 0.2 to 0.3 mg per liter of culture (33), significantly lower than the yield of disulfide-engineered DQ7Fc. The immunogenic potential of the long flexible linker is also uncertain. Lastly, KIH-based Fc-fusion proteins have offered a promising strategy to produce functional class II HLA antigens with increased yields (31), which can be coupled with nanoparticles to induce the formation of specific T-regulatory type 1 cells (58). Distinct from the KIH-based strategy, the disulfide-engineered dimeric DQ7Fc may have several potential advantages. It doesn't require "knob" and "hole" mutations within the Fc portion to achieve an increased yield. The protein is expected to fold into a more natural, antibody-like structure, replacing the two Fab arms with two DQ7 antigens. The doubled antigens may also increase the binding avidity to cognate T or B cell receptors on target cells.

In contrast to class II HLA molecules with α and β chains of comparable sizes, class I HLA molecules are heterodimers of a heavy chain (α chain) and a much smaller β2 microglobulin (β2m) with distinct structures and functions. Recently, Sgourakis' group has created disulfide-engineered class I HLA molecules, called “open MHC-I”, and added new insights about the dynamics between HLA conformation, peptide exchange, and protein complex stability (59). The mutations, G120C on the α chain and H31C on β2m, were at residues conserved across different HLA subtypes. The engineered proteins showed enhanced stability and were resistant to irreversible aggregation after peptide unloading. Interestingly, the covalently linked β2m allosterically promoted an open conformation in the heavy chain, allowing more efficient peptide exchange across a broad collection of HLA subtypes (59). This exciting technology is expected to facilitate peptide library screening and accelerate the discovery of pathogenic TCRs. Unlike our approach to class II HLA, the heavy chain and β2m of “open MHC-I” were first produced separately in *E. coli,* followed by refolding the complex in the presence of peptides. It will be interesting to know whether disulfide engineering can also increase the expression and production yield of recombinant class I HLA molecules in a mammalian cell culture system with or without a linked peptide. Conversely, interchain disulfide engineering may also affect peptide exchange in class II HLA molecules, further speeding up the peptide and TCR discovery for cancers, infections, and autoimmune diseases.

In summary, we conducted a proof-of-principle study of disulfide-engineered, dimeric HLA-DQ-Fc fusion proteins, focusing on their production, biochemistry, and potential application in targeting antibody-producing cells in AMR. The encouraging results support further development of this technology. In addition to the AMR-related application, future investigations can also be expanded to examine the interaction between disulfide-engineered class II HLA and disease-relevant TCRs and whether the same approach can be successfully applied to diverse subtypes of HLA-DQ, -DR, and -DP.

## Experimental procedures

### Reagents and resources

A list of reagents and resources used in this study and their identifiers were included in the online supporting information.

### Cell culture

CHO-S cells (Invitrogen) were grown in suspension in CHOgro Expression Medium (Mirus) in a shaking incubator at 37 °C until transfection for class II HLA protein production. IVD12 B cell hybridoma cells were obtained from ATCC and cultured in DMEM supplemented with glucose (4.5 g/L), 1 mM sodium pyruvate, 15% heat-inactivated fetal bovine serum, and antibiotics at 37 °C.

### Plasmids

The extracellular domains of HLA-DQ7 β chain (DQB1∗03:01) and α chain (DQA1∗05:01) were codon-optimized for mammalian cells (GenScript) and cloned into the pcDNA3.4 vector. The natural signal peptide sequence of the α chain (MILNKALMLGALALTTVMSPCGG) was replaced with the signal peptide sequence from azurocidin preproprotein (MTRLTVLALLAGLLASSRA) to enhance protein secretion into mammalian culture supernatant. For the β chain, the natural signal peptide sequence was retained. Either a placeholder peptide CLIP (PVSKMRMATPLLMQA) or other peptides to be tested and a (GGGSG)_2_ flexible linker were inserted between the signal peptide and β chain sequences; the latter was followed by a human IgG1 Fc fragment. All sequences encoding the wild type and variant DQ7Fc proteins were synthesized at GenScript.

### Protein expression and purification

Dimeric DQ7Fc recombinant proteins were first transiently expressed in CHO-S cells (Invitrogen). Healthy CHO-S cells with viability >97% and at a concentration of ∼4.0 × 10^6^ cells/ml were transfected with TransIT-PRO (Mirus) in Optimum Growth Flasks (Thompson) according to the manufacturer's recommendations. TransIT-PRO was premixed with plasmids for DQ7Fc β and α chains at a molar ratio of 1:2, with a final concentration of 1 mg total DNA per liter of culture. The Transfection kit enhancer was added immediately post-transfection at the recommended volumes. Cultures were kept at 32 °C for 7 days before harvest. The supernatant of the cell culture was collected by centrifugation at 5000*g* for 10 min at 4 °C, followed by filtration through a 0.22 μm regenerated cellulose membrane. The secreted proteins were purified by affinity chromatography using a MabSelect PrismA Protein A column (Cytia). Fc fusion proteins were eluted with 0.1 M sodium citrate (pH 3.0) and neutralized immediately with 1 M Tris-HCl (pH 9.0). Purified proteins were then exchanged to 1× PBS.

We used ion exchange chromatography to further purify ACEC. Q Sepharose fast flow resin (Cytia #17051001) was used to separate the major dimeric DQ7Fc from the minor aggregate and β chain-Fc dimer. Dimeric DQ7Fc was eluted with 250 mM NaCl in 20 mM Phosphate buffer, while aggregates and β chain-Fc dimer were eluted with higher NaCl concentration. The purity of eluted fractions was analyzed by non-reducing SDS-PAGE and analytical size exclusion chromatography (SEC; TSKgel G3000SWXL using 1XPBS as the elution buffer).

The reference protein, DQ7Fc(KIH), was produced based on the work by Serra *et al.* (31). Briefly, DNA sequences encoding the α chain (DQA1∗05:01) and β chain (DQB1∗03:01) of DQ7 were codon-optimized for mammalian cells (GenScript) and cloned into the pcDNA3.4 vector in frame with downstream sequences for human IgG1 Fc_knob (S350C/T362W) and Fc_hole (Y377C/T394S/L369A/Y435V), respectively. Endotoxin-free plasmids encoding the paired α and β chains were co-transfected at 1:1 into CHO cells using TransIT-PRO (Mirus), after which cells were cultured at 32 °C for 7 days before harvesting. DQ7Fc^KIH^ in the culture supernatant was purified by protein A chromatography and exchanged into phosphate-buffered saline (PBS) as described above.

### ELISA for protein quantification

Anti-DQ7 antibody purified from IVD12 B cell hybridoma cells was used to pre-coat 96-polystyrene-well microplates (Nunc Maxisorp, Thermo Fisher) at the final concentration of 0.3 μg/ml. The wells were washed and blocked with 5% bovine serum albumin in PBS/T (0.1%). Day 7 supernatant of transfected CHO-S cell culture or purified proteins were then added and incubated for 1 h at room temperature. The plates were washed again, and goat antibodies against human IgG, conjugated to horseradish peroxidase (Jackson Immuno Research), were added. After incubation for an hour at room temperature and subsequent washing, a chromogen-substrate solution (3,3′,5,5′-tetramethylbenzidine and hydrogen peroxide) was added. The reaction was stopped with sulfuric acid, and the optical density was measured at 450 nm using the Molecular Devices VersaMax Microplate reader.

### Protein electrophoresis

The protein samples (5 μg) were electrophoresed in 4 to 20% SDS-PAGE gels (Bio-Rad). For reducing conditions, samples were diluted 1:4 with 4x Laemmli sample buffer (Bio-Rad), supplemented with 10% 2-Mercaptoethanol (final concentration 2.5%), and then boiled at 100 °C for 5 min. Non-reducing conditions were assessed by diluting samples 1:4 with 4× sample buffer (100 mM Tris-HCl, pH 6.8, 40% glycerol, and bromophenol blue) without boiling and loading them directly onto the gel. Gel images were captured using a ChemiDoc MP imaging system (Bio-Rad). Subsequent analysis of gel images was conducted using ImageLab software (Bio-Rad) for quantification and characterization of protein bands.

### Western blot

For Western blot analysis, proteins were transferred onto nitrocellulose membranes following SDS-PAGE (Bio-Rad). The membranes were blocked with 5% BSA in PBS/T (0.1%) for 1 h and then incubated with one of the following antibodies: 1) 1:200 diluted IVD12 (purified in-house, 0.3 mg/ml), 2) 1:250 diluted SPV-L3 (0.2 mg/ml; Novus Biologicals), and 3) 1:10,000 diluted 1A3 (10 mg/ml, Leinco Technologies). After washing with PBS/T, membranes were incubated for 1 h with 1:5000 diluted IRDye 800CW goat anti-mouse (Licor). Finally, membranes were washed again using PBS/T and detected using Licor ODYSSEY.

### Bioinformatic analysis

The structure of HLA-DQ2 (PDB ID: IS9V) and -DQ8 (PDB ID: 1JK8) was visualized using the Mol∗ 3D viewer at rcsb.org (60). Sites for insertion of disulfide bridges were selected using the MODIP server (http://caps.ncbs.res.in/iws/modip.html). For analysis of the mutated positions for disulfide engineering, multisequence alignment files for HLA-DQA1 and -DQB1 protein sequences were downloaded from IPD-IMGT/HLA database (version 3.54 released in October 2023) and processed using an in-house script to generate a text file listing each allele and its protein sequence on each line. Graphical representations of the HLA-DQA1 and -DQB1 alignments were generated using weblogo with default parameters (61). Visualization of the surface exposure of mutated residues in HLA-DQ7 was performed using pHLA3D (38).

### FlowPRA assay

To verify the anti-DQ7 specificity of antibodies produced by IVD12 cells, FlowPRA assay was conducted using the FlowPRA Single Antigen HLA Class II - 4 Antibody Detection Test kit (One Lambda, West Hills, CA. #FL2HD04) on the AttuneNxT instrument (Invitrogen) following the manufacturers' instructions.

### Cell binding assay

IVD12 hybridoma cells (ATCC) were washed in DPBS and plated at 50,000 cells per well on a V-bottom, 96-well plate. After blocking with TruStain FcX (BioLegend) at 1:200 in staining buffer (1x PBS with 0.5% BSA, 2 mM EDTA), increasing amounts of DQ7Fc proteins was added to the buffer as indicated, or PBS was added as a negative control. After incubation at 4 °C for 20 min, cells were washed three times in buffer and stained with FITC-conjugated anti-human IgG at 1:100 each. After a second incubation at 4 °C for 30 min, cells were washed three times, resuspended in 200 μl of buffer, and analyzed by flow cytometry on the AttuneNxT instrument (Invitrogen).

### Flow cytometric complement-dependent cytotoxicity (CDC) assay

DQ7-specific B cell hybridoma cells (IVD12, ATCC) were washed in DPBS and plated at 50,000 cells per well on a V-bottom 96-well plate. Cells were pelleted by centrifugation at 1400 rpm and 4 °C for 4 min. Freshly thawed rabbit serum (One Lambda, #CDR-50) was diluted at 1:8 in DPBS. DQ7Fc proteins were added to the diluted rabbit serum at a concentration of 0.01 mg/ml followed by 1:2 serial dilutions to a titer up to 256. Rabbit serum with and without HLA-Fc was added to pelleted hybridoma cells at 45 μl/well and incubated at room temperature for 3 h in the dark. The cells were washed in 150 μl and then 200 μl staining buffer and then resuspended in 100 μl buffer containing 7-AAD diluted at 1:50 followed by flow cytometric analysis on the AttuneNxT instrument (Invitrogen).

### Data analysis and statistics

All data were from at least three independent experiments. For protein expression levels measured by ELISA, interpolated concentrations and 95% confidence intervals from the standard curve were reported from representative experiments. For antibody binding with titrated DQ7Fc proteins, means and standard deviations (SD) of the OD values were plotted against protein concentrations and fitted with the four-parameter logistic (4PL) regression model. For the CDC assay, means and SD of percent survival (7AAD-) of treated cells *versus* untreated cells were plotted against protein concentrations; CC50 was determined for each wild-type or variant protein by nonlinear regression using a variable slope model. All data analyses were performed using Prism version 9.2.0 (GraphPad Software, LLC).

## Data availability

All data pertaining to this study are contained within the article and supplemental data.

## Supporting information

This article contains supporting information.

## Conflict of interests

The authors declare the following financial interests/personal relationships which may be considered as potential competing interests.

X.A., C.L., and H.G. have financial interests in Antiger Therapeutics Inc. A patent application on Class II HLA with Inter-Chain Disulfide Bond has been filed in the US patent office (Inventor: C.L. and H.G.; Antiger Therapeutics Inc; US Provisional Application No. 63/613880).

## Supporting information

Supplementary Figure s
