# Supplementary figures and images for: Interchain disulfide engineering enables the efficient production of functional HLA-DQ-Fc fusion proteins
Source: J Biol Chem. 2024 Aug 8;300(9):107652. doi: 10.1016/j.jbc.2024.107652 (PMC11402769; doi:10.1016/j.jbc.2024.107652)

Supplemental Figure 1

A α chain

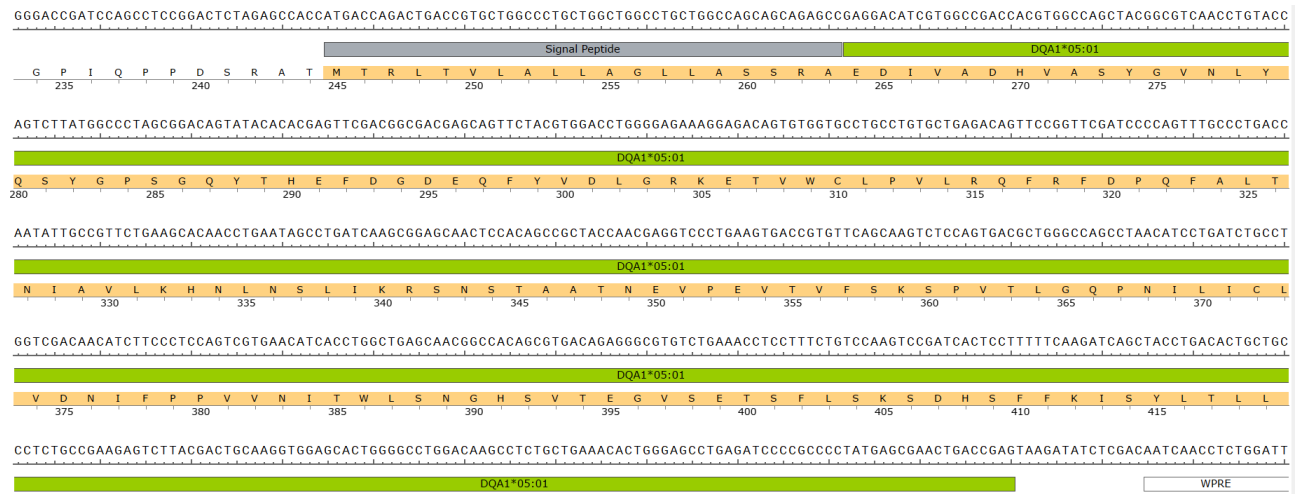

Supplemental Figure 1

B β chain

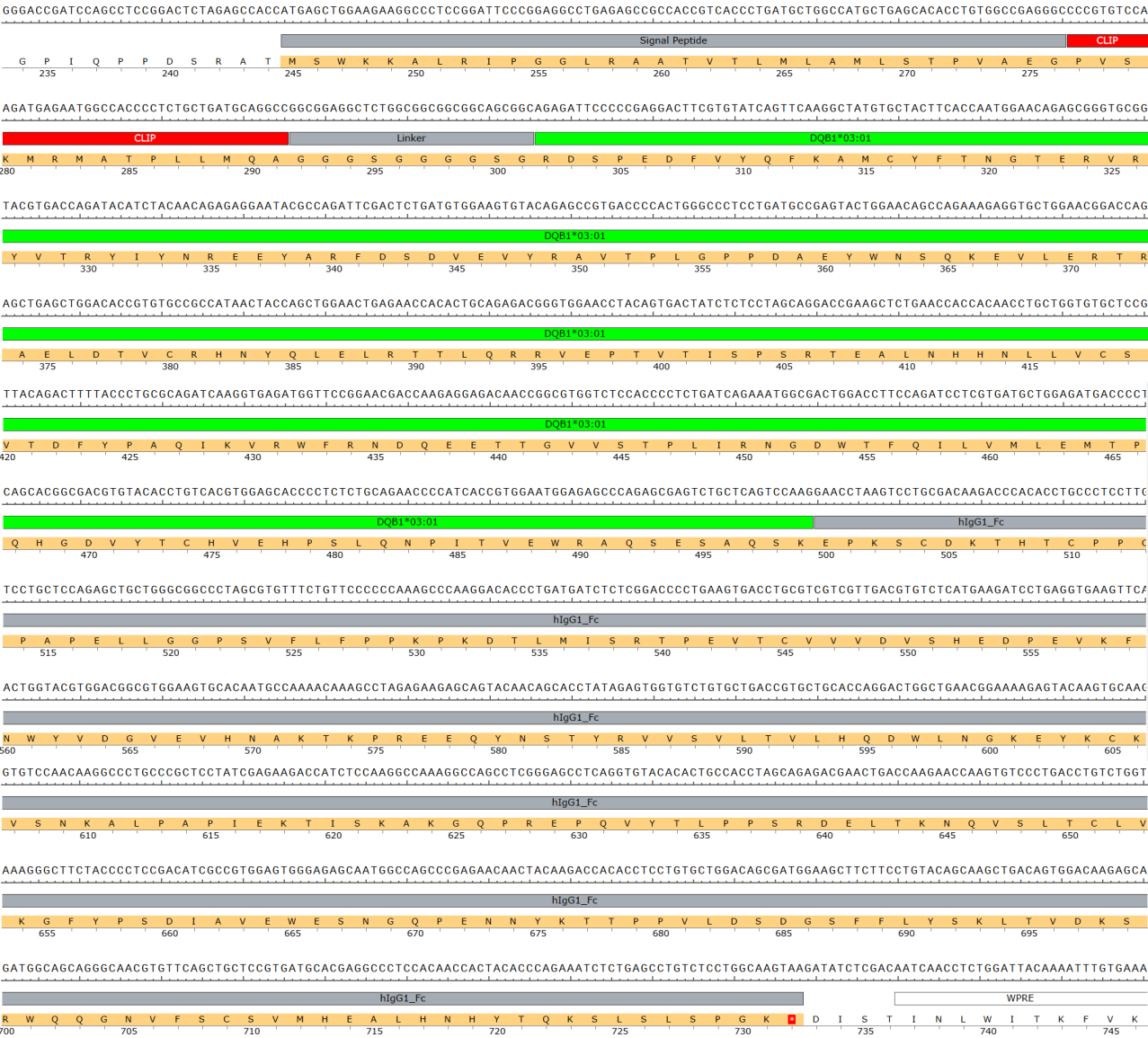

Supplemental Figure 2

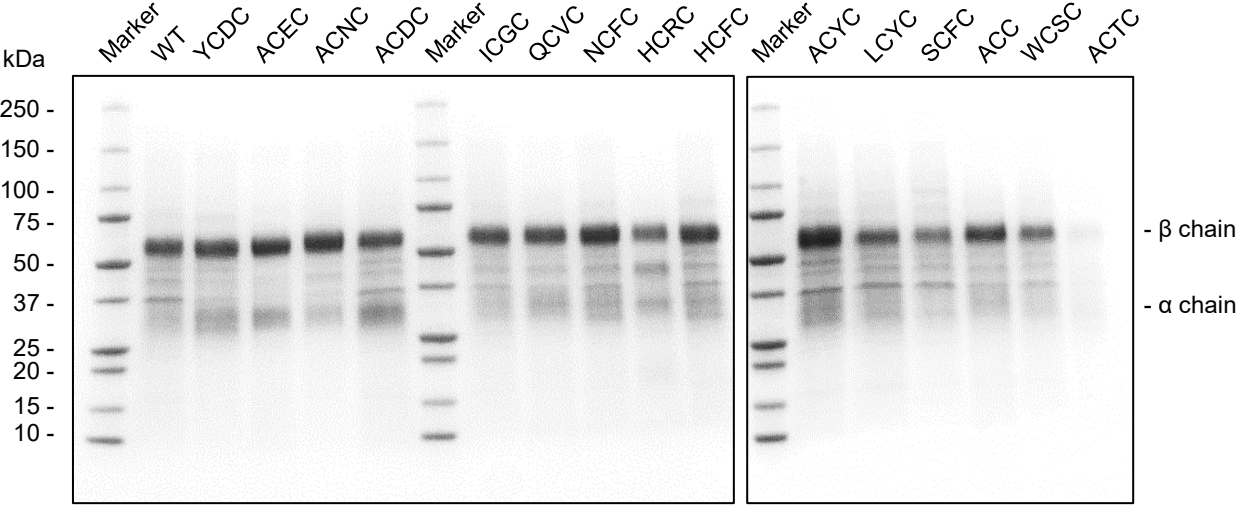

Supplement: Supplementary Figure — s [file mmc1.pdf]
