# Supplementary material for: Interchain disulfide engineering enables the efficient production of functional HLA-DQ-Fc fusion proteins
Source: J Biol Chem. 2024 Aug 8;300(9):107652. doi: 10.1016/j.jbc.2024.107652 (PMC11402769; doi:10.1016/j.jbc.2024.107652)
Supplement: Supporting information [file mmc2.docx]

# Title

Interchain Disulfide Engineering Enables Efficient Production of Functional HLA-DQ-Fc Fusion Proteins

# Authors

Xiamuxiya Aisihaer^1^, Hongjie Guo^1^, Chang Liu^1^

^1^Antiger Therapeutics Inc., 4340 Duncan Ave STE 284, St. Louis, MO 63110

# Correspondence authors

Chang Liu, [chang6@antigertherapeutics.com](mailto:chang6@antigertherapeutics.com); Hongjie Guo, [hguo@antigertherapeutics.com](mailto:hguo@antigertherapeutics.com).

# Running title

Class II HLA-Fc with interchain disulfide bond

# Keywords

Human leukocyte antigen, HLA-DQ, class II HLA, disulfide bond, Fc fusion protein, antibody-mediated rejection, immunotherapy

# Data availability

All data pertaining to this study are contained within the article and supplemental data.

# Supporting information

This article contains supporting information.

# Acknowledgments

This work was supported by the National Institute of Allergy and Infectious Disease (R43AI174328; C.L. and H.G).

# Supporting information

# Key experimental reagents and resources

| **REAGENT OR RESOURCE** | **SOURCE** | **IDENTIFIER** |
| --- | --- | --- |
| Experimental Models: Cell Lines |  |  |
| CHO-S cells | Gibco | Cat#R80007 |
| IVD12 mouse B cell hybridoma cells, HLA DQ3(7)-specific (IgG1) | ATCC | Cat#HB-144 |
| Antibodies and Viability Dyes |  |  |
| Peroxidase AffiniPure™ Goat Anti-Human IgG, Fcγ fragment specific | Jackson | Cat#109-035-190 |
| FITC-conjugated goat anti-human IgG Fc | Jackson | Cat#109-095-170 |
| FITC-conjugated goat anti-mouse IgG | BioLegend | Cat#405305 |
| PE-conjugated goat anti-mouse IgG (clone Poly4053) | BioLegend | Cat#405307 |
| Rat anti-mouse CD16/32 antibody (TruStain FcX, clone 93) | BioLegend | Cat#101320 |
| 7AAD viability staining solution | Invitrogen | Cat#00-6993-50 |
| Bacterial Strains |  |  |
| One-Shot DH5alpha chemically competent *E. coli* | Invitrogen | Cat#C404010 |
| Chemicals, Enzymes, Buffers, and Media |  |  |
| CHOgro Complex Formation Solution | Mirus | Cat#MIR6210 |
| L-Glutamine Solution – 100 mL | Mirus | Cat#MIR6240 |
| CHOgro Expression Medium | Mirus | Cat#MIR6200 |
| CHOgro® Titer Enhancer | Mirus | Cat#MIR6220 |
| Poloxamer188 10%Solution | Mirus | Cat#6230A |
| TransIT-PRO® Transfection Reagent | Mirus | Cat#MIR5740 |
| Penicillin-Streptomycin (5,000 U/mL) | RPI | Cat#12608776 |
| DMEM | ATCC | Cat#30-2002 |
| Fetal bovine serum (FBS, heat-inactivated 30min at 56°C) | ATCC | Cat#30-2021 |
| Bovine serum albumin (BSA) | Omnipur | Cat#9048-46-8 |
| EDTA | Sigma | Cat#03690 |
| Protein A Agarose Resin (50% suspension) | Cytiva | Cat#17549802 |
| Sodium Citrate Tribasic Dihydrate | Millipore sigma | Cat#567446 |
| Tris base | Sigma-Aldrich | Cat#RDDoo8 |
| 10x Phosphate-buffered saline (PBS) | Sigma | Cat#P5493-4L |
| Dulbecco's Phosphate-buffered saline (DPBS, Ca-, Mg-) | ATCC | Cat#30-2200 |
| DMSO, Hybri-Max™, sterile-filtered | Sigma | Cat#D2650 |
| Rabbit serum complement | One Lambda | Cat#CDR-50 |
| Sodium Pyruvate Solution | Sigma-Aldrich | Cat#S8636-100ml |
| Software and Database |  |  |
| ApE-A Plasmid Editor v2.0.53c | M. Wayne Davis | N/A |
| SignalP 5.0 server | www.cbs.dtu.dk | N/A |
| The Immune Epitope Database (IEDB) | www.iedb.org | N/A |
| The IPD-IMGT/HLA Database | www.ebi.ac.uk | N/A |
| FlowJo v10.7.1 | Becton Dickinson | N/A |
| pHLA3D 2.0 | www.phla3d.com.br | N/A |
| GraphPad Prism v9 | GraphPad Software | N/A |
| RCSB Protein Data Bank | [www.rcsb.org](http://www.rcsb.org) | N/A |

# Supplemental Figure legends

## Supplemental Figure 1. Sequences for soluble dimeric DQ7Fc.

***A***, the extracellular domains of HLA-DQ7 α chain (DQA1*05:01) and ***B***, β chain (DQB1*03:01) were codon-optimized for mammalian expression and cloned into the pcDNA3.4 vector. The natural signal peptide (SP) sequence of the α chain (MILNKALMLGALALTTVMSPCGG) was replaced with the signal peptide sequence from azurocidin preproprotein (MTRLTVLALLAGLLASSRA) to enhance protein secretion into mammalian culture supernatant (upper panel). For the β chain (lower panel), the natural signal peptide sequence was retained. A placeholder peptide CLIP (PVSKMRMATPLLMQA) and a linker were inserted between the signal peptide and β chain sequences; the latter was followed by a human IgG1 Fc fragment.

## Supplemental Figure 2. SDS-PAGE analysis of dimeric DQ7Fc proteins under a reducing condition.

Wild type (WT) and disulfide-engineered DQ7Fc variants were purified from the supernatants by protein A chromatography and analyzed by SDS-PAGE under a reducing condition. The β chain of ~50 kDa was observed across all variants; the α chain of ~25 kDa was also observed with variable intensities.
